# Technical note: preliminary surgical experience with a new implantable epicranial stimulation device for chronic focal cortex stimulation in drug-resistant epilepsy

**DOI:** 10.1007/s00701-024-06022-0

**Published:** 2024-03-22

**Authors:** Volker A. Coenen, Nadja Jarc, Martin Hirsch, Peter C. Reinacher, Bernhard J. Steinhoff, Thomas Bast, Andreas Schulze-Bonhage, Bastian E. A. Sajonz

**Affiliations:** 1 Department of Stereotactic and Functional Neurosurgery, Medical Center of Freiburg University, Breisacher Straße, 64–79106 Freiburg, Germany; 2 Epilepsy Center, Neurocenter, Medical Center of Freiburg University, Breisacher Straße, 64–79106 Freiburg, Germany; 3 Medical Faculty of Freiburg University, Freiburg, Germany; 4 Fraunhofer Institute for Laser Technology (ILT), Aachen, Germany; 5 Kork Epilepsy Center, Kehl-Kork, Germany; 6 Center for Deep Brain Stimulation, Medical Center of Freiburg University, Breisacher Straße, 64–79106 Freiburg, Germany

**Keywords:** Drug-resistant epilepsy, Neuromodulation, Chronic epicranial focal cortex stimulation, Surgical experience

## Abstract

**Purpose:**

This study is to report some preliminary surgical considerations and outcomes after the first implantations of a new and commercially available implantable epicranial stimulation device for focal epilepsy.

**Methods:**

We retrospectively analyzed data from clinical notes. Outcome parameters were as follows: wound healing, surgery time, and adverse events.

**Results:**

Five patients were included (17–52 y/o; 3 female). Epicranial systems were uneventfully implanted under neuronavigation guidance. Some minor adverse events occurred. Wound healing in primary intention was seen in all patients. Out of these surgeries, certain concepts were developed: Skin incisions had to be significantly larger than expected. S-shaped incisions appeared to be a good choice in typical locations behind the hairline. Preoperative discussions between neurologist and neurosurgeon are mandatory in order to allow for the optimal coverage of the epileptogenic zone with the electrode geometry.

**Conclusion:**

In this first small series, we were able to show safe implantation of this new epicranial stimulation device. The use of neuronavigation is strongly recommended. The procedure is simple but not trivial and ideally belongs in the hands of a neurosurgeon.

**Supplementary Information:**

The online version contains supplementary material available at 10.1007/s00701-024-06022-0.

## Introduction

Neurostimulation for drug-refractory epilepsy follows distinct principles of overall brain excitability reduction like vagus nerve stimulation [6], responsive lesion site stimulation [14], or network hub modulation like ANT DBS [4]. Focal epicranial stimulation is a new addition to this armamentarium [10, 12], which in first studies has shown promising effects in focal drug-resistant epilepsy in scenarios where resective surgery is either no option or simply not wanted by the patient [8, 11, 14]. Such a system (EASEE®, Precisis, Heidelberg, Germany) is now CE-marked and commercially available in Europe. The system should, in principle, be minimally invasive and well tolerated due to its epicranial positioning.

Neurostimulation of the epileptic focus directly targets the region of seizure generation, unlike stimulation types targeting seizure spread and general cortical excitability. The EASEE device uses both intermittent bursts of high frequency stimulation and DC-like stimulation with cathodal pulses delivered via the central electrode of the array above the hyperexcitable cortex. Either approach has been shown to be effective in reducing epileptic activity and seizure generation [10]. Epicranial electrode positioning has several rationales. First, unlike external transcutaneous stimulation, e.g., with tDC stimulators, antiseizure treatment with an implanted and invisible device is non-stigmatizing and can be performed in an automated manner avoiding potential adherence problems [1]. Second, unlike intracranial placement of electrodes and stimulator as performed with responsive neurostimulation, the risk profile can be assumed to be better, the positioning of the electrode externally of the skull avoids typical complications like intracerebral bleeding (4.7%) and device infections (9.4%) [3]. Third, placement of electrodes below the galea reduces the distance to the brain as well as leak currents between stimulation electrodes, leading to a reduction of the stimulus amplitude needed to induce similar effects on neuronal firing rates in the underlying cortex by a factor of 4 [15]. The epicranial localization of the electrode array thus allows to use less power for applying currents, with positive effects on battery life of the stimulator.

To our knowledge, surgical considerations on *implantation strategies, sequence, feasibility*, and *tolerability of this system* have not been reported so far. We here present our early surgical experience in the commercial application phase. We report on a series of patients who underwent implantation at our institution and discuss our workflow, surgical and anatomical considerations, and short-term (surgical) outcomes.

## Methods and material

### Ethics

This case series follows the tenets of the declaration of Helsinki. Written informed consent was obtained from the patients who received a draft version of this paper.

We selected five consecutive patients admitted for implantation after the device became commercially available. We retrospectively analyzed data from outpatient records, clinical notes and surgical reports. Outcome parameters for this small case series were as follows: wound healing, surgery time and any adverse event reporting in the notes.

### Implantation procedure

After induction of general anesthesia, the patient was positioned on the OR table and the head pinned in a Mayfield clamp. Care was given to positioning the patient to allow a single-staged approach with the ability to tunnel along the lateral neck. Limited shaving, draping and administration of antibiotics (Cefuroxime, 1.5 g, Sandoz, Austria). After incision and preparation of the skull, subcutaneous tunneling to the pectoral region was performed. A subcutaneous pectoral pocket was prepared to accommodate the internal pulse generator (IPG) which was fixed with non-dissolvable sutures to the pectoralis fascia. The electrode array’s position was navigated (Intellect, Stryker, Freiburg, Germany), and the electrode then fixed to the skull with seven bone screws (Vigomed, Höchberg, Germany). After the fixation impedance, checks were performed. Wound closure was done in multiple fascial layers. Patients went to the normal ward and were discharged after 3 days. Wound stitches were removed after 10–14 days.

## Results

**Fig. 1:**
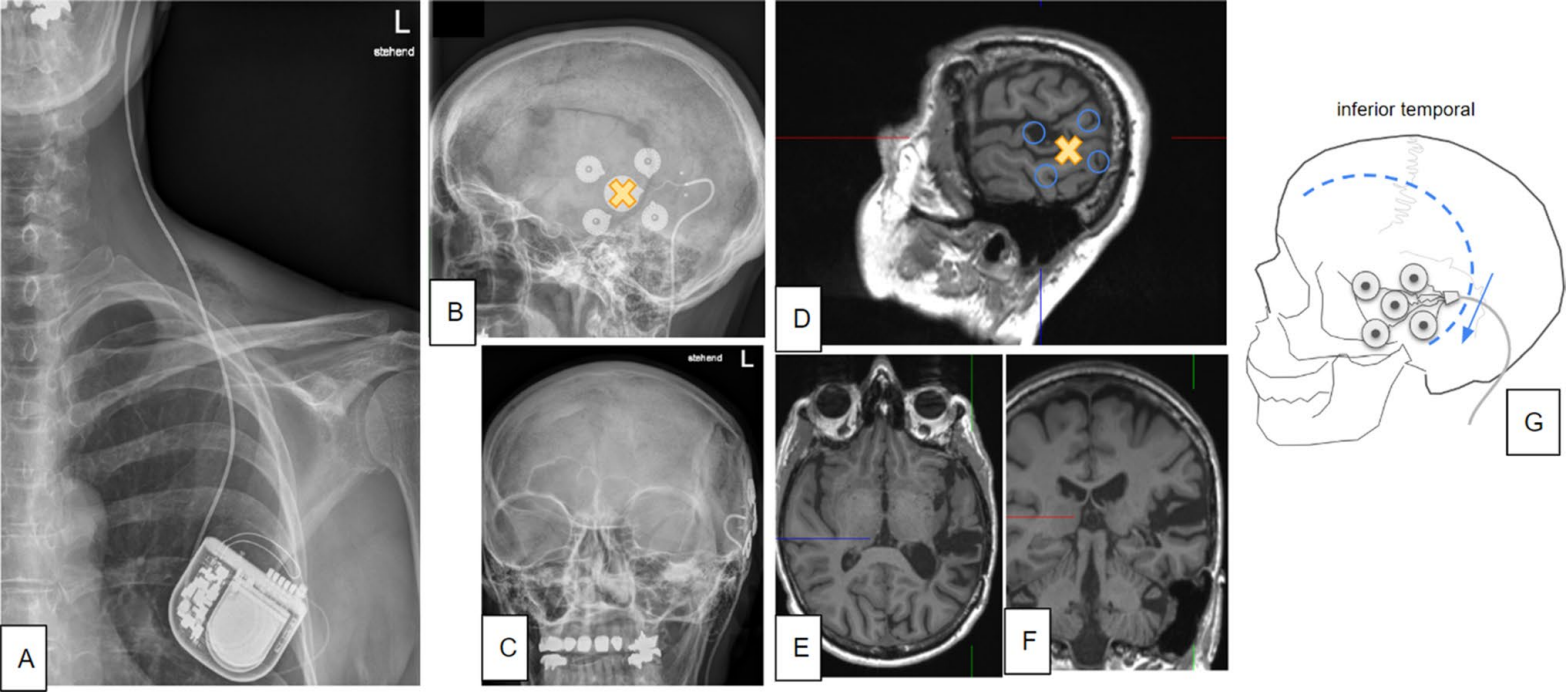
Case 1. **A–C** X-rays of a fully implanted EASEE® system. (**B**) shows previous craniotomy. **D–F** T1 weighted MRI showing lesion. X indicates the planned position of central (cathodal) contact. **G** Cartoon depiction of electrode position. Curvilinear incision (scar from previous craniotomy) reaches far enough behind the ear to allow subcutaneous tunneling of electrode (blue arrow)

**Fig. 2:**
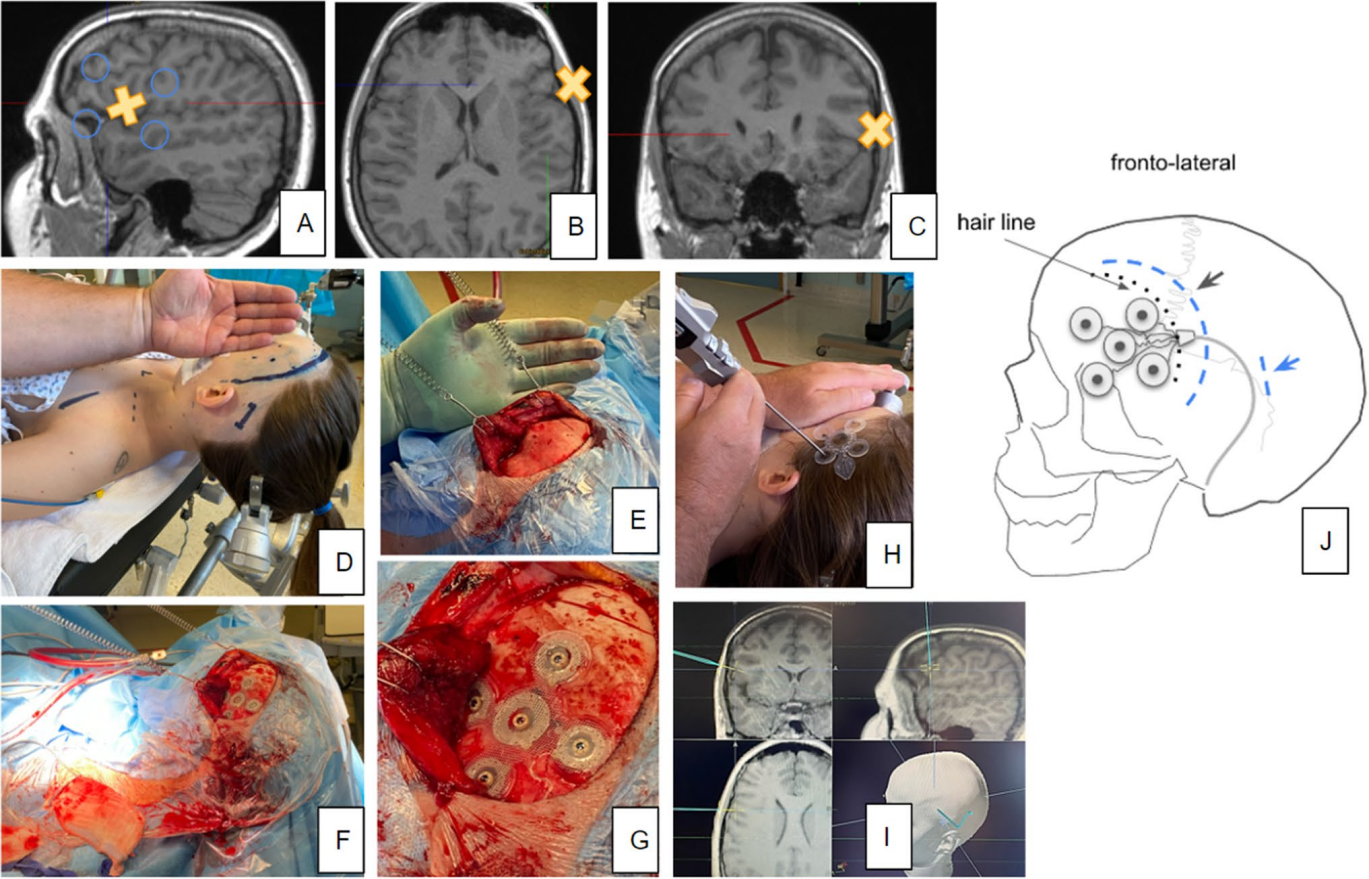
Case 4. **A–C** T1 weighted MRI showing NO lesion. X indicates the planned position of central (cathodal) contact. **D** The patient is placed supine, and the head is pin fixed. **E–G** Preparation steps include cutting of the temporalis muscle and placing the electrode array. **H, I** Neuronavigation. **J** A cartoon depiction of electrode position. The curvilinear incision behind the hairline, additional incision behind the ear (blue) for subcutaneous tunneling of the electrode

Five patients (17–52 y/o; 3 female) were included. All patients besides patient 4 received commercial implantation of the device outside of study activities. Only patient 4 was implanted as part of the EASEE4YOU study. Patients underwent uneventful implantation of the stimulation device under general anesthesia. Operation time varied between 46 and 81 min (mean 66 min). Follow-up times were 150 to 206 days (179 ± 23 days). There were no serious adverse events: One patient (no 5) developed a small subgaleal hematoma yet showed uneventful wound healing. Patient 1 reported some discomfort at the pectoral stimulator site related to the mere presence of the stimulator itself. Patient 3 reported some discomfort when lying on her occipitally situated electrode implant. Detailed patient characteristics and surgical outcomes are shown in Table 1. Figures 1 and 2 (Cases 1 and 4, respectively) exemplify the individual surgical strategies taken.

**Table 1:** Patient characteristics and outcomes

No	age	sex	lesion	follow up [days]	surgery duration	stim. active	comment	wound healing
1	62	f	left temporal cavernoma, venous dysplasia, resection cavity,	206	66 min	yes	some discomfort at stimulator site pectoral	primary
2	24	m	right occipital/ parietal FCD	193	73 min	yes	-	primary
3	21	f	left parietal/occipital gliotic scar tissue	186	63 min	yes	some pressure pain when lying on occiput	primary
4	17	f	none; EEG focus left inferior frontal gyrus and anterior and superior temporal gyrus	160	81 min	yes	EASEE4YOU study*	primary
5	22	m	FCD, right precentral and suppl. motor area	150	46 min	yes	small subgaleal hematoma, later some minimal seroma, later entirely resolved	primary

All patients received a commercial clinical implant besides patient 4 who was implanted as part of the EASEE4YOU study (*DRKS00031722)

## Discussion

We here present our early experience with a new commercially available and CE-marked epicranial stimulation device (EASEE®, Precisis, Heidelberg, Germany). Our patients tolerated the implantation procedure well, with only minimal adverse events (cf. Table 1). Implantation of the entire system is straightforward. However, the system has certain idiosyncrasies which should be respected to allow for a successful and efficacious implantation.

### Incision planning

**Fig. 3:**
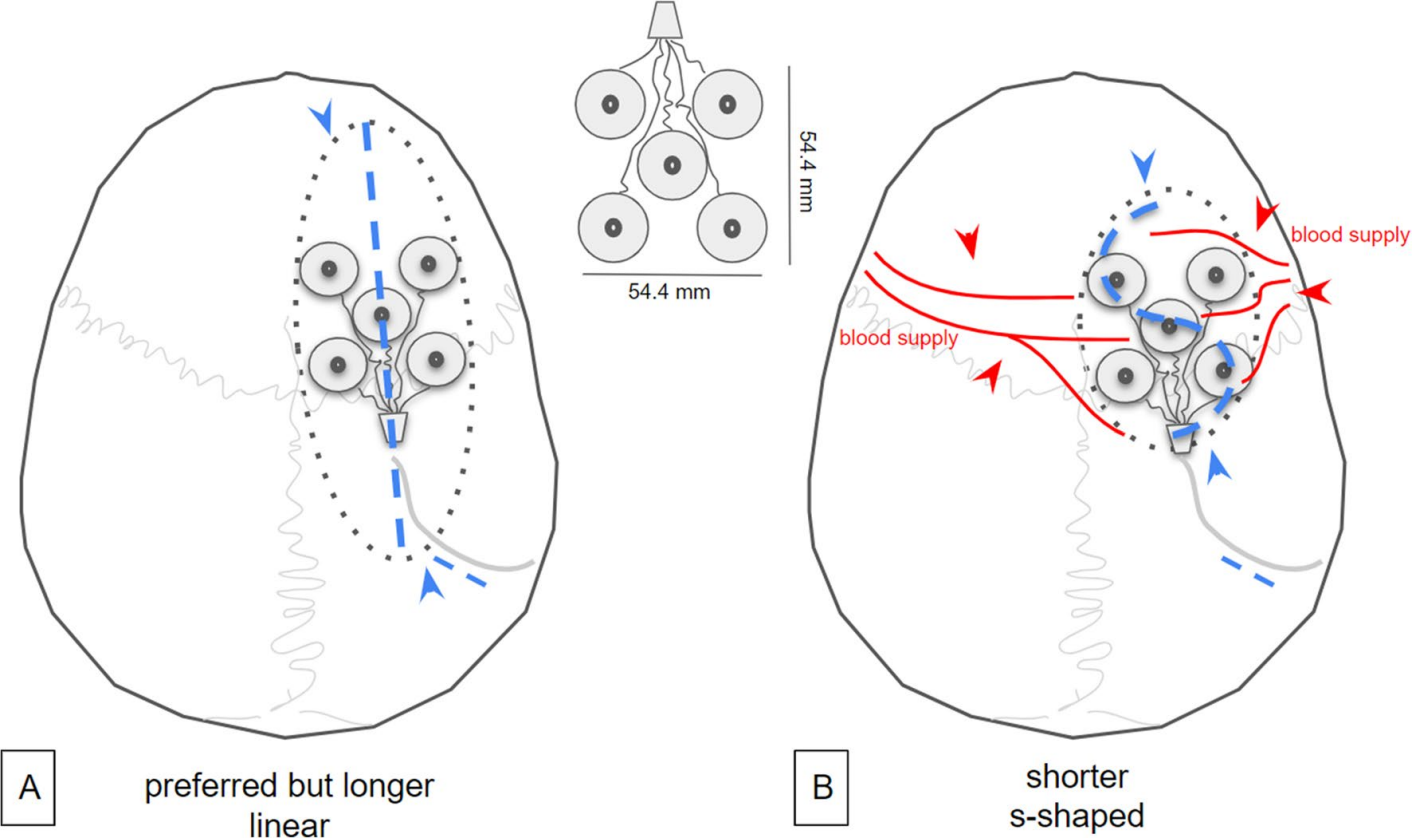
Electrode geometry (inset) and possible incisions.** A** Linear cut needs to be longer to allow for wide enough exposure of the cranium (dotted line, arrows) to fit the electrode array. **B** An S-shaped incision is preferable. It is shorter and allows for a shorter wound and broader exposure of the cranium (dotted line, arrow). The orientation of the S should respect the vascular supply (red arrows)

**Fig. 4:**
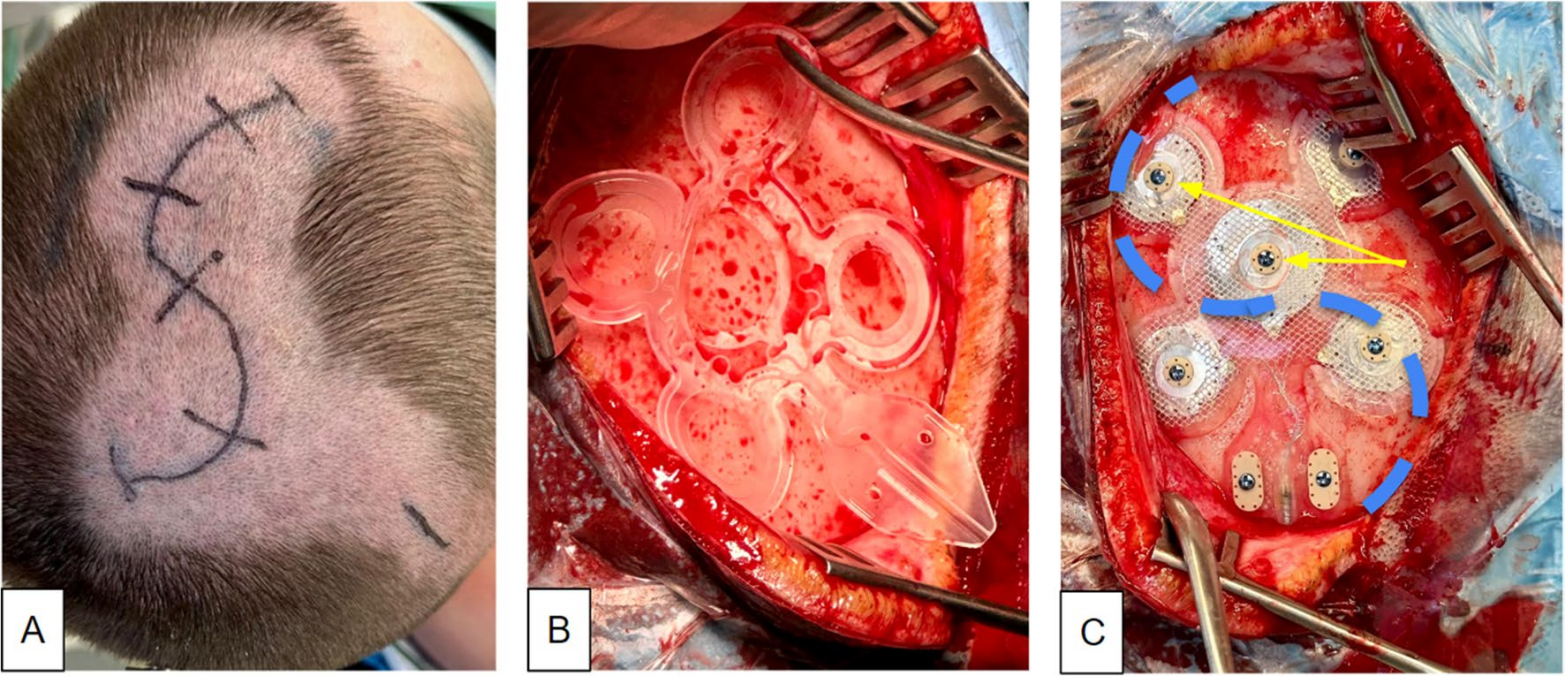
Case 5 with right prefrontal positioning of the electrode array. **A** Limited shaving and marking of S-shaped skin incision.** B** Fitting of sterile electrode mold. **C** final electrode position with bone screws (two of seven marked with yellow arrows) in place, skin incision marked (blue)

Skin incision needs to be centered on the epicranial electrode to allow for tight fitting to the skull and screw fixation of the seven bone screws. In principle, such incision would be performed as a straight cut. However, a straight cut would, in most instances, be rather long to accommodate for the electrode size (54.4 × 54.4 mm^2^ Fig. 3). For most cases, we therefore chose to use an S-shaped incision (Fig. 3B, 4) accommodating for the best ratio between incision length and yield of epicranial exposure. It is important to align the cut so that the two flaps are well-supplied with blood [9] (Fig. 3). We have successfully used this type of incision already in clinical studies [11] and found them to be well tolerated and healing well. A skin flap which covers the whole electrode would in principle have been possible. We found such an approach unnecessarily invasive unless a position in proximity to the hairline (fronto-lateral position) was needed (Fig. 2). The incision further needs to include a region behind the ear in order to accommodate electrode tunneling subcutaneously to the pectoral region. Sometimes, an additional incision might be necessary (Figs. 2 D and J and 4A). It is important to inform the patient about the skin incision size. In advertisements, the company has shown the electrode to be *simply* slipped under the scalp through a small cut, which may lead to an underestimation of the surgical procedure.

### Electrode positioning and fixation

The array electrode needs to have a tight fit to the skull to assure minimal impedance and minimize leak currents [5, 11]. As a preparational step, the period needs to be entirely scraped away. Petechial bleedings from the tabula externa of the bone might prevent a fully dry situation. In these instances, we have used a large diamond burr in order to shave the outer layer of the bone, which typically stops bleeding. The drilling might sometimes be necessary to reduce unevenness of the bone which will later underlie the electrode. Bone wax cannot be applied since it could act as an insulation. Plastic material used for bone reconstruction is a contraindication for using the system. It will likewise serve as an insulator. If previous cranial surgery occurred, we recommend performing pre-implantation X-rays in order to look for alloplastic material and metal cranial fixations which might also present a contraindication. Before the electrode fixation, the tunneling of the distal cable to the pectoral region should be performed. Neuronavigation is used to achieve the optimal electrode position (Fig. 2). The electrode is finally secured with seven bone screws (Vigomed, Höchberg, Germany; Figs. 4C). After the connection to the IPG, regular current flow is checked through repeated intraoperative impedance measurements.

### Neuronavigation

We have started to use neuronavigation in this indication already during the study phase of implantations [11]. It is our experience that the center cathodal contact of the pseudo-Laplacian electrode should be no further than 1 cm away from the planned implantation and later stimulation point. Most neuronavigation systems warrant the use of a pin fixation of the patient’s head. This is of importance because patients need to be conscientious about postoperative discomfort/pain at pin sites. As in any epilepsy surgery, a joint interpretation of localizing information for the center and borders of the epileptogenic zone between the neurologist and the neurosurgeon about the warranted optimal electrode position (Figure S1_supplement) is necessary for treatment success.

### Implantation of the internal pulse generator (IPG)

To allow for a single staged procedure, the patient should initially be positioned so that subcutaneous tunneling along the lateral neck is possible (Fig. 2D). The implantation of the IPG is performed as known from DBS surgery. We either make a 3-cm-wide straight incision 1–2 cm below and parallel to the clavicle. In female patients, we sometimes use an aslant cut from the anterior axillary line which—if available—follows a skin fold in the direction towards the jugulum. This incision might be cosmetically advantageous. The patient should be made aware of the size of the stimulator.

### Wound healing and infection rate

In our center, we ensure that prior to surgery, no urinary tract infections or systemic infections (determined with C-reactive protein, leukocyte count, fever) are present. Empirically, we prophylactically administer a short course of antibiotics up to 24 h after surgery (Cefuroxime (Sandoz GmbH, Austria), 1.5 g, i.v.; 1st dose 30 min prior to incision, dose 2 after 8 h, and dose three after 18 h) which is our analogue practice in VNS implantations. We have so far not seen any infections. This might be due to the short mean operation time of around 1 h. Moreover, we are dealing with a younger patient population as opposed to the Parkinson’s DBS population which might reduce the infection rate. Realistically, the expected infection rate will range somewhere between VNS [2, 13] and DBS surgery [7] and should in larger (and later reported) groups range somewhere between 1 and 6%. Infections are multifactorial and centers should rule out systemic infections and try to keep operating time short.

### Limitations

The limitations of our case series are the small number of patients and the rather short follow-up times. However, we are to a certain extent basing our evaluation and recommendations on our experience from previous trial surgery of the past years (EASEE, EASEE II, PIMIDIS trials [11]), where no late complications occurred following device implantations for follow-up periods of up to 36 months so far. Due to the short follow-up time, outcome data of stimulation cannot be presented, and we refer to the pertinent literature.

## Conclusion

Implantation of this epicranial stimulation device can be performed with a low complication rate and is in principle well tolerated. Exact placement using a neuronavigation offers the best chances for an optimal spatial coverage of the epileptogenic area with fields of stimulation. Due to the size of the electrode, skin incisions must be large enough to accommodate the electrode array. The array is not simply “slipped under the scalp,” and the procedure is therefore (in the original sense of the term) not minimally invasive. However, with an epicranially residing electrode, the entire approach is less invasive than any other long-term neuromodulatory approach.

## Supplementary Information

Below is the link to the electronic supplementary material.

Supplementary file1 (PNG 188 KB)

## Data Availability

Not applicable.
