# Supplementary figures and images for: Technical note: preliminary surgical experience with a new implantable epicranial stimulation device for chronic focal cortex stimulation in drug-resistant epilepsy
Source: Acta Neurochir (Wien). 2024 Mar 22;166(1):145. doi: 10.1007/s00701-024-06022-0 (PMC10957708; doi:10.1007/s00701-024-06022-0)

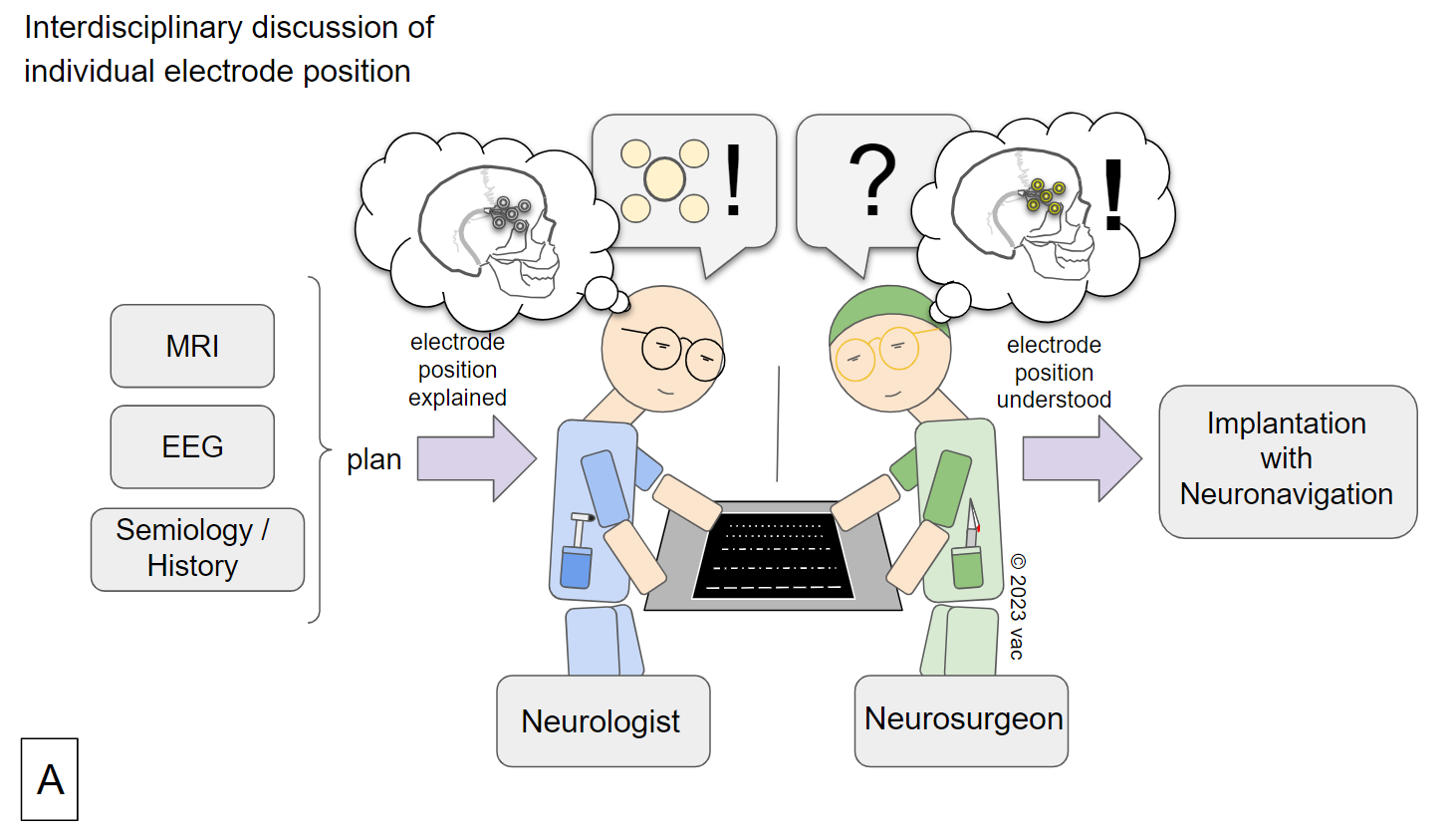

Supplement: Supplementary file 1 — Supplementary file1 (PNG 188 KB) [file 701_2024_6022_MOESM1_ESM.png]
